# Clinical relevant Bruton’s X-linked tyrosine kinase deficiency in a female with extreme X-chromosome inactivation

**DOI:** 10.1186/s13223-025-01002-0

**Published:** 2025-12-02

**Authors:** Sundus M. NoorSaeed, Abdulaziz S. Alrafiaah, Manar Alghamdi, Adam J. Shapiro, Christine McCusker

**Affiliations:** 1 Department of Pediatrics, Faculty of Medicine, King Abdulaziz University, Jeddah, Kingdom of Saudi Arabia; 2 Department of Pediatrics, Division of Allergy and Clinical Immunology and Dermatology, Montreal Children’s Hospital, Montreal, QC Canada; 3 Department of Pediatrics, College of Medicine, Majmaah University, Majmaah, 11952 Saudi Arabia; 4 Department of Pediatrics, Division of Respiratory Medicine, Research Institute of the McGill University Health Centre, Montreal, QC Canada

**Keywords:** Agammaglobulinemia, BTK, X-chromosome inactivation, XLA, Immunodeficiency, Bronchiectasis, Bruton’s, IVIG, CVID, Chronic sinusitis

## Abstract

**Background:**

X-linked agammaglobulinemia (XLA) is an inborn error of immunity resulting from mutations in the *BTK* gene. It is an X-linked inherited disease that almost exclusively affects males, while females are usually carriers of the disease. However, certain genetic conditions can lead to XLA disease expression in females. We report a 14-year-old girl who was diagnosed with XLA from a pathogenic *BTK* variant and skewed X chromosome inactivation (XCI).

**Case presentation:**

A 14-year-old female Ukrainian refugee was referred to the respirology clinic at the Montreal Children’s Hospital with an abnormal chest radiograph found during her immigration medical screening process in Canada. She was reported to be previously well until the age of 11 years when she was diagnosed with pneumonia following SARS-CoV-2 infection. She subsequently developed recurrent pneumonias, persistent productive cough, and chronic sinusitis with polyposis. Laboratory investigations showed severely reduced serum immunoglobulin G, A, and M concentrations of 0.78 g/L, < 0.10 g/L, and 0.21 g/L, respectively. Flow cytometry for lymphocyte subsets showed a complete absence of circulating B cells in peripheral blood. Next-generation sequencing panel for inborn errors or immunity revealed a heterozygous pathogenic variant in *BTK* (c.862C > T, p.Arg288Trp). Analysis of XCI revealed markedly skewed inactivation (99:1), resulting in predominant expression of the mutant pathogenic BTK allele. These findings support the clinical diagnosis of X-linked agammaglobulinemia manifesting in a female patient due to skewed XCI.

**Conclusions:**

Female carriers of pathogenic mutations in BTK may develop clinically important disease as a result of extreme random X inactivation.

## Background

X-linked agammaglobulinemia (XLA), also known as Bruton’s disease, is an inborn error of immunity first described in 1952 by Ogden Bruton. Pathogenic variants in the *BTK* gene, leading to arrested B-cell maturation and differentiation, near absence of circulating mature B cells, and markedly reduced serum immunoglobulin levels [1]. XLA results from pathogenic variants in the BTK gene, which is located on the X chromosome. Because males are hemizygous for the X chromosome, they are primarily affected [2]. The disease may arise from maternally inherited variants or de novo mutations. When the variant is maternally inherited, each son has a 50% risk of being affected, and each daughter has a 50% chance of being a carrier. Female carriers are generally asymptomatic due to balanced X-chromosome inactivation [3]. Certain genetic conditions can lead to *BTK* allele expression in females, including skewed X-Chromosome Inactivation (XCI)**,** homozygosity, and chromosomal translocation or Turner Syndrome (Monosomy X)^(3)^. We report a 14-year-old girl who was diagnosed with XLA secondary to skewed X-Chromosome inactivation.

## Case presentation

A 14-year-old female Ukrainian refugee was referred to the respirology clinic at the Montreal Children’s Hospital with an abnormal chest radiography characterized by consolidations in the lingula and left lower lobe. Computed tomography (CT) of the chest showed cylindrical bronchiectasis with tree-in-bud opacities in the lingula and left lower lobe. Chronic maxillary sinusitis with nasal polyposis was seen on CT imaging of the sinuses. Investigations for mycobacterial infection and cystic fibrosis were negative. Sputum and sinus cultures were repeatedly positive for *Haemophilus parainfluenzae*, while spirometry was normal (FEV_1_ at 106% predicted).

Her past medical history revealed that she is the product of a twin pregnancy, born to non-consanguineous, White parents without a family history of confirmed inborn errors of immunity. She reported a relatively well childhood without recurrent, invasive, or bizarre infections. Around age 7 years, she developed recurrent otitis media and two episodes of cervical adenitis. At age 11, she was diagnosed with pneumonia and SARS-CoV-2 infection. Subsequently, she developed recurrent pneumonias, some requiring hospitalization for intravenous antibiotics, a persistent wet productive cough, and chronic sinusitis with polyposis.

Her family history was relevant for frequent sinopulmonary infections in her father, who was never formally evaluated and is deceased. The mother and the patient’s twin sister were generally healthy. Our patient had received routine vaccinations in Ukraine; including: HBV, DTP, Polio, Hib, MMR, and Bacille Calmette-Guérin vaccination, without identified adverse events.

Her examination showed normal growth parameters, absence of dysmorphic features, absence of tonsils, and lymph nodes were not palpable.Laboratory investigations showed severely decreased serum immunoglobulin G, A, and M concentrations of 0.78 g/L, < 0.10 g/L, and 0.21 g/L, respectively. Post-vaccine serologic responses were non-protective for tetanus, diphtheria, rubella, and hepatitis B. Flow cytometry for lymphocyte subsets showed normal T cells but a complete absence of circulating B cells in peripheral blood. [Table 1] A next-generation sequencing primary immunodeficiency panel from Blueprint revealed a heterozygous pathogenic variant in *BTK* (NM_000061.2: c.862C > T, p.Arg288Trp, ClinVar ID: 11,366). Parental genetic testing was not performed; therefore, the possibility of a de novo variant could not be confirmed. X-linked, missense variant occurs in exon 10 of *BTK* and is not present in population databases (gnomAD: no frequency). It has been previously observed in individuals with X-linked agammaglobulinemia [4] and segregates with the disease in related individuals. Experimental studies have shown this variant affects *BTK* function [5] by disrupting the p.Arg288 amino acid residue, and other variants that disrupt this residue are pathogenic [6–8].

**Table 1:** Laboratory results for the patient

*Parameter*	
Absolute lymphocyte count (ALC)	5.67
CD3 (cells/μL) absolute number CD3%	5260 96
CD4 absolute number CD4%	2693 49
CD8 absolute number CD8%	2369 43
CD19 absolute number CD 19%	9 0
CD16/56 absolute number CD16/56%	135 2
*Serum immunoglobulins*	
IgG (g/L)	0.78
IgM (g/L)	0.21
IgA (g/L)	< 0.10
IgE (IU/mL)	8
*Vaccine response titers*	
Diphtheria [IU/mL]	< 0.01
Tetanus antibody [iu/ml]	< 0.01
Hepatitis B Surface Antibody [IU/L]	0.2
Rubella IgG	0.1

The patient’s twin sister underwent immunological evaluation and was shown to have normal serum immunoglobulin levels. Since the proband was an affected female, we conducted X-Chromosome Inactivation (XCI) analysis, which demonstrated markedly skewed X-Chromosome inactivation (99:1), above the normal 90% ratio of inactive to active X. These findings support predominant expression of the mutant allele and the clinical diagnosis of X-linked agammaglobulinemia (XLA) with a heterozygous, pathogenic variant in *BTK*.

**Fig. 1:**
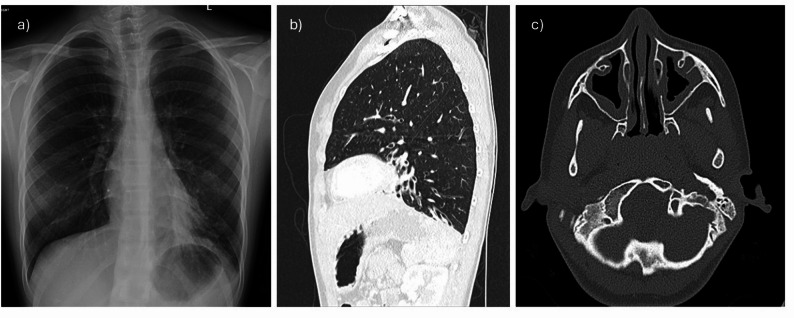
Clinical radiology studies on the female patient with X-linked agammaglobulinemia. **a** Posterior-anterior chest radiography with left lower lobe opacity at presentation, **b** Sagittal view of chest computed tomography showing cylindrical bronchiectasis in the lingula and left lower lobe, **c** Computed tomography of the head showing bilateral mucosal thickening of the maxillary sinuses

This patient received intravenous immunoglobulin (IVIG) replacement (0.5 g/kg every 4 weeks) and, there times per week oral azithromycin prophylaxis, 10 mg/kg orally for frequent pulmonary exacerbations. There was some reduction of her productive cough, and her rhinosinusitis has also improved after six months of therapy. Her IgG levels were normal with replacement therapy (Fig. 1).

## Discussion

In females, one of the two X chromosomes is typically inactivated at random in each somatic cell during early embryonic development. However, when this inactivation becomes skewed, favoring wild-type X-Chromosome inactivation, female carriers of X-linked disease alleles can manifest symptoms of X-linked recessive diseases, which are otherwise predominantly seen in males [9–13].

In view of this patient’s clinical presentation and agammaglobulinemia, the differential diagnosis includes autosomal recessive forms of the disease (such as defects in IGHM, IGLL1, CD79A/B, PIK3R1, PIK3CD), as well as, more rarely, XLA. However, in light of the genetic findings, with the absence of features consistent with Turner Syndrome, the leading differential diagnosis was XLA due to skewed XCI. Rare cases have been reported in females with non-random inactivation of the normal X chromosome resulting in functional hemizygosity and leading to a clinical phenotype that is indistinguishable from that seen in affected males [2]. Takada and colleagues first reported female with XLA through a *BTK* mutation [Table 2]. Her father had been diagnosed with XLA in early childhood due to recurrent infections. Both the girl and her father lacked BTK protein expression in their monocytes. Genetic analysis of the proband revealed complete inactivation of her normal (maternal) X chromosome, leaving the paternal X chromosome (with the disease-causing *BTK* variant) active in blood and oral mucosa, leading to disease manifestation [14]. In another report by Garcia-Prat and colleagues, a 71-year-old woman who was initially diagnosed with common variable immunodeficiency(CVID) was found to carry a hypomorphic *BTK* variant (Tyr418His). Lyonization analysis revealed skewed XCI, with 80% of BTK expression derived from the mutant allele [15]. The timing of diagnosis may be variable, even in affected males with delayed diagnosis after age 10 years and some in adulthood, in approximately 10% of males with BTK pathogenic variants.

**Table 2:** Summary table of previously reported female cases of XLA

	P1 (Takada et al.)	P2 (Garcia-Prat et al.)	P3 (Our patient)
Age	10-month-old girl	71-year-old woman	14-year-old girl
Onset	2 months	35 years	7 years
Clinical presentation	Frequent respiratory infections, otitis media	Recurrent respiratory tract infections, otitis media, and bronchiectasis Hodgkin’s lymphoma	Recurrent otitis media, cervical adenitis, recurrent pneumonias, persistent wet cough, chronic sinusitis with polyposis
Genetic variant	Heterozygous mutation in the first single base pair of intron 11 (G > A) of the BTK gene	Heterozygous missense Hypomorphic BTK variant (Tyr418His) c.1252 T > C/p.Tyr418His	Heterozygous pathogenic BTK variant c.862C > T, p.Arg288Trp
X chromosome inactivation assay results	The maternal X chromosome was exclusively inactivated in mononuclear cells and oral mucosal cells. Non-random inactivation of the X chromosome in this patient was caused by unknown mechanisms other than XIST	Lyonization analysis showed XCI with 80% of BTK expression coming from the mutant allele. In gDNA analysis revealed balanced allelic representation, while cDNA showed a clear imbalance favoring the mutant allele. This was precisely quantified using high-coverage NGS-based deep amplicon sequencing, revealing a T/C allele ratio of 20:80, confirming skewed XCI at the expression level	Skewed XCI (99:1), with BTK expression mostly from the mutant allele

In girls with X-inactivation syndrome, the direction and extent of X-chromosome inactivation (XCI) skewing may affect disease severity in conditions such as hemophilia B, dyskeratosis congenita, Duchenne muscular dystrophy, myotubular myopathy, and Fabry disease [16]. However, there is limited documentation on XCI-mediated XLA and how its clinical presentation may relate to the degree of skewed XCI. As in our case, the relatively late onset of symptoms has been observed in previously reported cases of XLA in females, where the age of diagnosis ranged from 10 months to 35 years of age [Table 2]. The reasons for the clinical variability in presentation are unknown but may be related to the amount of residual antibody production in patients with later presentations.

## Conclusion

In female patients with agammaglobulinemia, *BTK* variants may induce XLA through skewed XCI. Absence of circulating B cells should prompt genetic evaluation for XLA, regardless of patient sex. Presentation is variable, and consequences of delayed diagnosis include chronic lung disease, which carries a poorer overall prognosis.

## Data Availability

No datasets were generated or analysed during the current study.

## Abbreviations

XLA: X-linked agammaglobulinemia
CVID: Common variable immunodeficiency
XCI: X chromosome inactivation
IVIG: Intravenous immunoglobulin

## References

[1] Bruton OC. Agammaglobulinemia. Pediatrics. 1952;9(6):722–8. 14929630

[2] Smith CIE, Berglöf A. X-Linked Agammaglobulinemia. In: Adam MP, Feldman J, Mirzaa GM, Pagon RA, Wallace SE, Amemiya A, editors. GeneReviews(®). Seattle (WA): University of Washington, Seattle. Copyright © 1993–2025, University of Washington, Seattle. GeneReviews is a registered trademark of the University of Washington, Seattle. All rights reserved.; 1993

[3] Basta M, Pandya AM. Genetics, X-Linked Inheritance. StatPearls, editor. Treasure Island (FL): StatPearls Publishing; 2023 2023 May 1.

[4] Fiorini M, Franceschini R, Soresina A, Schumacher RF, Ugazio AG, Rossi P, et al. BTK: 22 novel and 25 recurrent mutations in European patients with X-linked agammaglobulinemia. Hum Mutat. 2004;23(3):286. 14974089 10.1002/humu.9219

[5] Tzeng SR, Pai MT, Lung FD, Wu CW, Roller PP, Lei B, et al. Stability and peptide binding specificity of Btk SH2 domain: molecular basis for X-linked agammaglobulinemia. Protein Sci. 2000;9(12):2377–85. 11206059 10.1110/ps.9.12.2377 PMC2144513

[6] Plebani A, Soresina A, Rondelli R, Amato GM, Azzari C, Cardinale F, et al. Clinical, immunological, and molecular analysis in a large cohort of patients with X-linked agammaglobulinemia: an Italian multicenter study. Clin Immunol. 2002;104(3):221–30. 12217331 10.1006/clim.2002.5241

[7] Conley ME, Mathias D, Treadaway J, Minegishi Y, Rohrer J. Mutations in btk in patients with presumed X-linked agammaglobulinemia. Am J Hum Genet. 1998;62(5):1034–43. 9545398 10.1086/301828 PMC1377085

[8] Conley ME, Broides A, Hernandez-Trujillo V, Howard V, Kanegane H, Miyawaki T, et al. Genetic analysis of patients with defects in early B-cell development. Immunol Rev. 2005;203:216–34. 15661032 10.1111/j.0105-2896.2005.00233.x

[9] Aral B, de Saint Basile G, Al-Garawi S, Kamoun P, Ceballos-Picot I. Novel nonsense mutation in the hypoxanthine guanine phosphoribosyltransferase gene and nonrandom X-inactivation causing Lesch-Nyhan syndrome in a female patient. Hum Mutat. 1996;7(1):52–8. 8664901 10.1002/(SICI)1098-1004(1996)7:1<52::AID-HUMU7>3.0.CO;2-R

[10] Azofeifa J, Voit T, Hübner C, Cremer M. X-chromosome methylation in manifesting and healthy carriers of dystrophinopathies: concordance of activation ratios among first-degreefirst degree female relatives and skewed inactivation as cause of the affected phenotypes. Hum Genet. 1995;96(2):167–76. 7635465 10.1007/BF00207374

[11] Clarke JT, Greer WL, Strasberg PM, Pearce RD, Skomorowski MA, Ray PN. Hunter disease (mucopolysaccharidosis type II) associated with unbalanced inactivation of the X chromosomes in a karyotypically normal girl. Am J Hum Genet. 1991;49(2):289–97. 1678247 PMC1683291

[12] Nisen PD, Waber PG. Nonrandom X chromosome DNA methylation patterns in hemophiliac females. J Clin Invest. 1989;83(4):1400–3. 2564852 10.1172/JCI114028 PMC303834

[13] Parolini O, Ressmann G, Haas OA, Pawlowsky J, Gadner H, Knapp W, et al. X-linked Wiskott-Aldrich syndrome in a girl. N Engl J Med. 1998;338(5):291–5. 9445409 10.1056/NEJM199801293380504

[14] Takada H, Kanegane H, Nomura A, Yamamoto K, Ihara K, Takahashi Y, et al. Female agammaglobulinemia due to the Bruton tyrosine kinase deficiency caused by extremely skewed X-chromosome inactivation. Blood. 2004;103(1):185–7. 12958074 10.1182/blood-2003-06-1964

[15] Garcia-Prat M, Batlle-Masó L, Parra-Martínez A, Franco-Jarava C, Martinez-Gallo M, Aguiló-Cucurull A, et al. Role of skewed X-chromosome inactivation in common variable immunodeficiency. J Clin Immunol. 2024;44(2):54. 38265673 10.1007/s10875-024-01659-z

[16] Sun Z, Fan J, Wang Y. X-Chromosome inactivation and related diseases. Genet Res (Camb). 2022;2022:1391807. 35387179 10.1155/2022/1391807 PMC8977309

